# Unusual piezochromic fluorescence of a distyrylpyrazine derivative crystals: phase transition through [2 + 2] photocycloaddition under UV irradiation

**DOI:** 10.1038/s41598-021-81562-4

**Published:** 2021-02-02

**Authors:** Young-Jae Jin, Hyosang Park, Byung-Chun Moon, Jae Hong Kim, Wang-Eun Lee, Chang-Lyoul Lee, Giseop Kwak

**Affiliations:** 1 Department of Polymer Science and Engineering, School of Applied Chemical Engineering, Kyungpook National University, 1370 Sankyuk-dong, Buk-ku, Daegu, 702-701 Korea; 2 School of Chemical Engineering and Technology, Yeungnam University, 214-1 Dae-dong, Gyeongsan, 712-749 Gyeongbuk Korea; 3 Reliability Assessment Center for Chemical Materials, Korea Research Institute of Chemical Technology (KRICT), 141 Gajeong-ro, Yuseong-gu, Daejeon, 305-600 Korea; 4 Advanced Photonics Research Institute (APRI), Gwangju Institute of Science and Technology (GIST), 1 Oryong-dong, Buk-gu, Gwangju, Korea

**Keywords:** Organic chemistry, Materials science

## Abstract

The piezochromic fluorescence (FL) of a distyrylpyrazine derivative, 2,3-diisocyano-5,6-distyrylpyrazine (DSP), was investigated in this study. Depending on the recrystallization method, DSP afforded two different crystals with green and orange FL emission. The orange color FL emission crystal (O-form) was easily converted to the green color FL emission one (G-form) by manual grinding. The G-form was also converted to a slightly different orange color FL emission crystal (RO-form) by a weak UV irradiation. When the RO-form was ground again, the G-form was regenerated. The FL colors changed between the G- and RO-forms over several ten times by repeated mechanical grinding and UV irradiation. The FL, UV–visible, ^1^H-NMR and XRD results showed that the O (or RO)-to-G transformation induced by mechanical stress results from the change of degree of molecular stacking from dense molecular stacking structure to relatively loose molecular stacking structure, whereas the G-to-RO reconversion by UV irradiation results from return to dense molecular stacking structure again due to lattice movement (lattice slipping) allowed by photocycloaddition in solid-state.

## Introduction

The π-conjugated organic molecules have been extensively studied as key materials for opto-electronic device applications owing to their excellent and various functionalities in solid state^1–3^. These functionalities strongly depend on the molecular packing structures of π-conjugated organic molecules. Therefore, it is very important to understand the effects (roles) of self-assembly and electronic interactions between the molecules on their opto-electronic properties. Intermolecular interactions, such as π–π interactions, hydrogen bonding, and van der Waals force are very weak, but play a critical role in determining the intermolecular structures and opto-electronic properties^4–6^. Controlling the weak intermolecular interactions as desired would be of particular interest in sensor applications.

Recently, metachromatic π-conjugated chromophores or fluorophores have attracted lots of attention due to their highly advanced functions. Many types of metachromatic materials show significant optical changes in response to heat, light, solvents, surfactants, and mechanical stress. Among them, the materials whose fluorescence (FL) respond to mechanical stress like shearing, grinding, and elongation, which are said to have piezochromic FL (PCFL), are particularly interesting. The original FL emission of piezochromic material is changed by external physical stress and exclusively restored through heating or recrystallization^7–12^. Araki et al. firstly reported PCFL using a terpyridine derivative. The blue emission was changed to green emission by pressure and recovered by thermal annealing^13–16^. Regarding molecular design aspect, the introduction of intermolecular hydrogen bonding was critical factor for achieving the PCFL. Similarly, other research groups have also developed liquid–crystal like highly PCFL-active π-conjugated fluorophores^17–22^. In all the cases, PCFL occurs through phase transition triggered by pressure and heat under the control of weak intermolecular interactions.

**Scheme 1:**
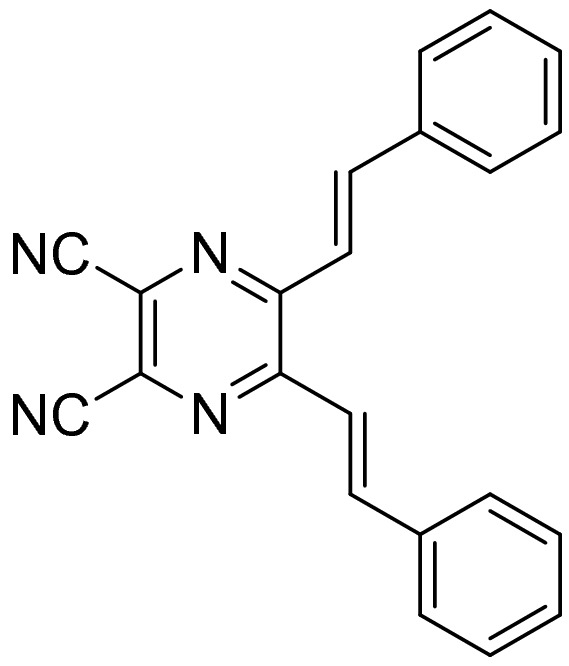
Chemical structure of DSP.

In this study, we report a unique PCFL-active metachromatic material, which recover its original FL emission by UV irradiation unlike the restoration of original color by thermal annealing or recrystallization. Recently, Kim, one among the authors, synthesized a polymorphic crystal compound of 2,3-diisocyano-5,6-distyrylpyrazine (DSP in Scheme 1)^23,24^. This compound exhibited two different types of crystal morphology. One type of crystal was obtained by recrystallization from a mixture of tetrahydrofuran (THF) and acetonitrile (v/v = 1:1). The other type of crystal was obtained by recrystallization from benzene. It is discovered that two crystals exhibited not only different FL colors, but also different photochemical reaction behaviors. These differences resulted from different molecular stacking structure of two crystals. The crystal obtained from the THF/acetonitrile mixed solvent showed the orange color emission with the FL emission maximum at 564 nm, while the crystal obtained from benzene showed the green color emission with the FL emission maximum at 523 nm. The orange color FL emission crystal (O-form) was readily transformed to a green color FL emission crystal (G-form) through manual grinding. Then, the G-form was converted to a slightly different orange color FL emission crystal (RO-form, here, R represents the recovery) by UV-irradiation. And when the RO-form was ground again, the G-form was completely restored. This PCFL behavior was highly reversible for several ten times and reproducible. Herein, we describe the PCFL mechanism in detail and suggest a new molecular design based on our findings.

## Results and discussion

**Figure 1:**
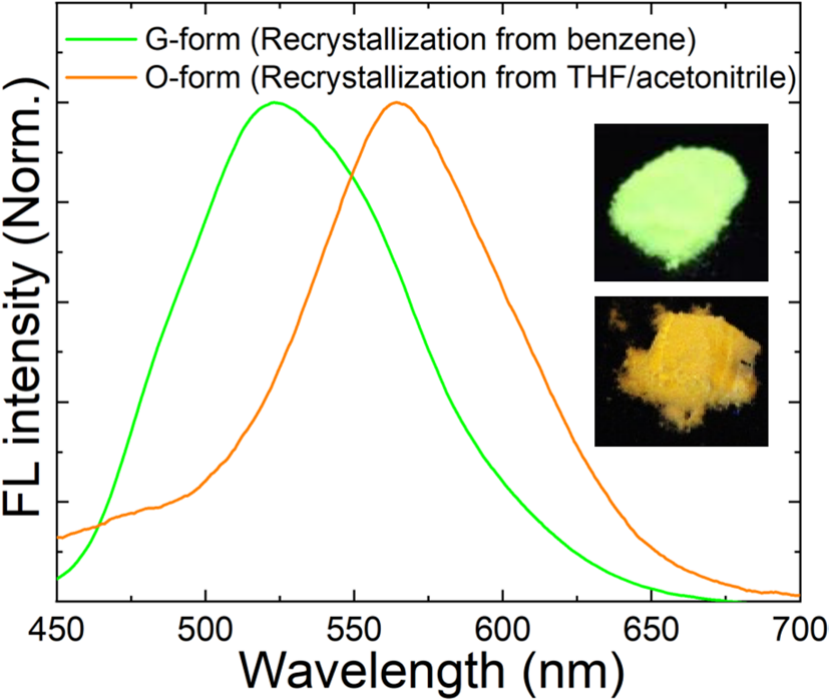
FL emission spectra and photographs of DSP crystals obtained by recrystallization from the 1:1 (v/v) mixture of THF/acetonitrile (O-form) and benzene (G-form).

**Figure 2:**
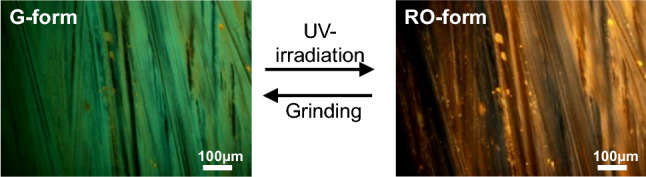
FL microscope images of DSP crystals in G- and RO-forms.

**Figure 3:**
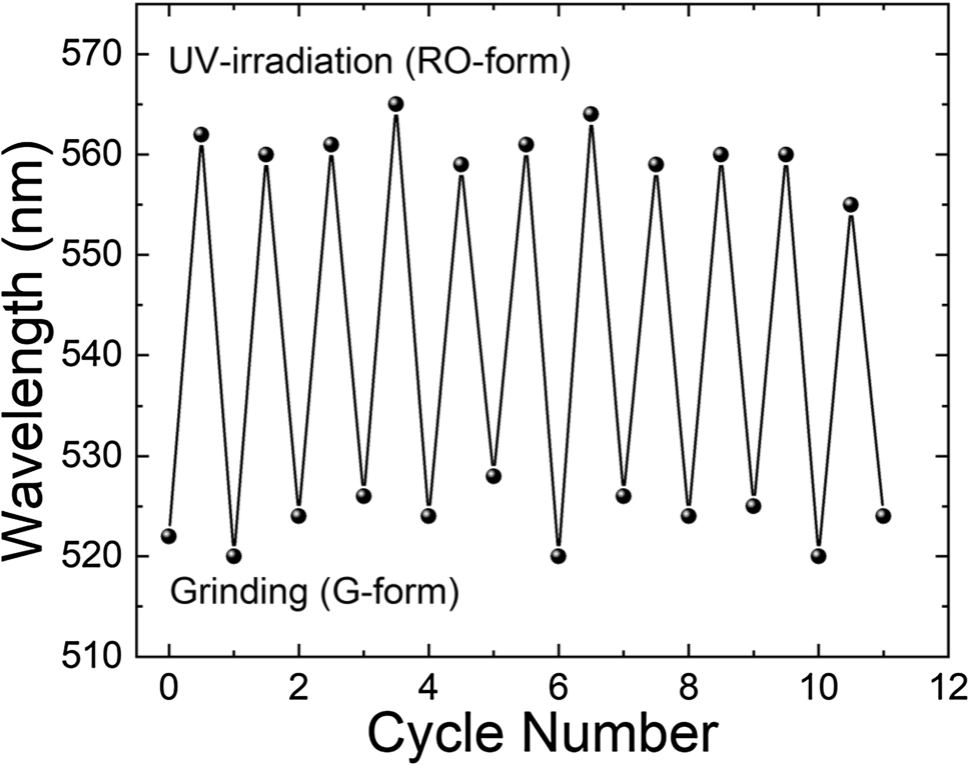
FL emission wavelength change of DSP crystals during repeated grinding and UV-irradiation cycles.

The DSP crystal obtained by recrystallization from a mixture of THF and acetonitrile (v/v = 1:1) showed orange FL emission (O-form shown in Fig. 1). When the crystal was manually ground using a mortar and/or pestle, the orange FL emission was changed to green emission (G-form shown in Fig. 1), the same as the color of crystal obtained by recrystallization from benzene. Therefore, it was assumed that a certain phase transition occurred due to mechanical stress, which resulted in the crystal structure change. Surprisingly, when the G-form was irradiated with UV light (365 nm, 2.5 J/cm^2^) for ~ 2 min, the orange color FL was readily recovered. However, the recovered FL color was slightly different from the original FL color of the O-form, as later described in detail. It is referred as the RO-form hereafter. Figure 2 shows the optical microscope images of G- and RO-forms with different FL colors. Similar to the O-form, when the RO-form was slightly ground, the G-form was regenerated. Subsequently, when irradiated under a UV lamp at a slightly higher power (12.5 J/cm^2^), the RO-form was more readily recovered within 3 s. This FL interconversion is quite different from the conventional PCFL accompanied by heating or recrystallization; hence, it can be considered as a new mechanism for PCFL behavior. Moreover, this FL interconversion was repeated over several ten times without any bleaching or discoloring (Fig. 3). Thus, the PCFL between RO- and G-forms was highly reversible and reproducible under a long-term operation.

**Figure 4:**
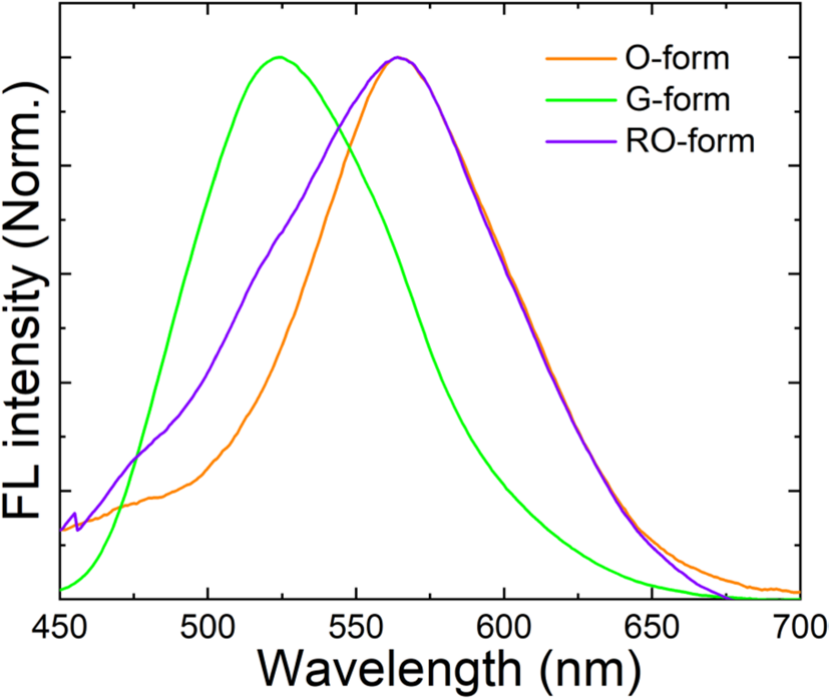
FL emission spectra of DSP crystals during the O → G → RO change.

**Figure 5:**
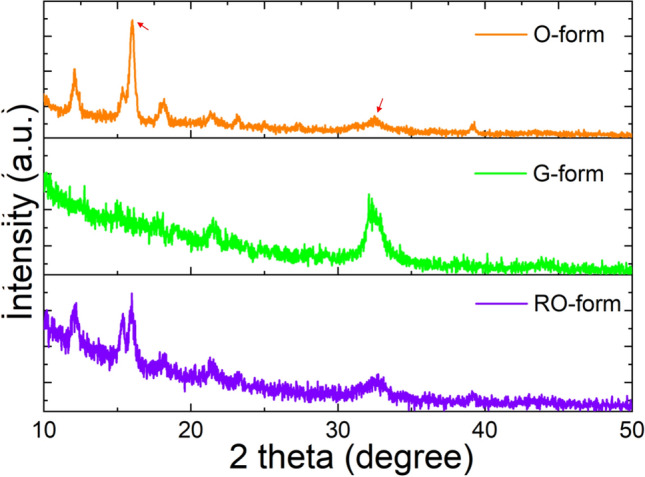
X-ray diffraction (XRD) patterns of DSP crystals in O-, G- and RO-forms.

Figure 4 represents the FL emission spectra change of DSP crystals in the solid state during the O → G → RO form change. The O- and RO-forms show similar FL emission with the maximum FL wavelength (λ_FL, max_) of 564 nm, whereas the λ_FL, max_ of the G-form was 523 nm. Moreover, the FL quantum yields (FLQYs) of O-, G-, and RO-forms in solid state were also measured and compared to each other. The FLQY of G-form was ~ 0.13%, which is about 2.5 times larger than that of O-form (~ 0.05%), and became smaller again (~ 0.07%) in RO-form. The ^1^H-NMR spectra of O- and G-forms were acquired by dissolving two different DSP crystals in chloroform (CDCl_3_). Both crystals showed a doublet at 7.67–7.72 ppm due to the vinylene protons and a multiplet at 7.40–7.50 ppm due to the phenyl ring protons (Fig. S1). These ^1^H-NMR spectra indicate that the O- and G-crystals have same chemical structure in isolated solution state, even though they exhibit different FL emission spectra in solid state. This resulted from the polymorphism of DSP, which is induced by a slight difference (~ 0.11 Å) in the interlayer *c*-axis distance between two crystals^23,24^. Since the O- and G-form have same crystal structure (monoclinic) and lattice parameters except for slight difference in the interlayer *c*-axis distance, therefore, it is expected that diffraction peaks in O-form will be similar as those of G-form in the XRD experiment. The O-form showed exactly same diffraction peaks at 2θ = 16.0°, 21.6°, 32.6° and 43.9°, observed in G-form, but showed additional diffraction peaks at 2θ = 12.1, 15.3, 18.1, 23.2 and 39.2°. (Fig. 5). Also, the ratios of diffraction peak intensity at 16.2° and 32.6° (marked by arrow) are different in the G- and O-form. It suggests that O-form has a different molecular stacking structure from G-form. The shorter *c*-axis distance in the O-form due to lattice contraction induced dense molecular stacking by enhanced the intermolecular π–π interaction^23^. Thus, the O-form presents more red-shifted FL emission than G-form. Therefore, the FL emission change from O- to G-form by manual grinding can be attributed to a crystal-to-crystal phase transition between two crystals with different molecular stacking structures by an external driving force, such as mechanical stress.

**Figure 6:**
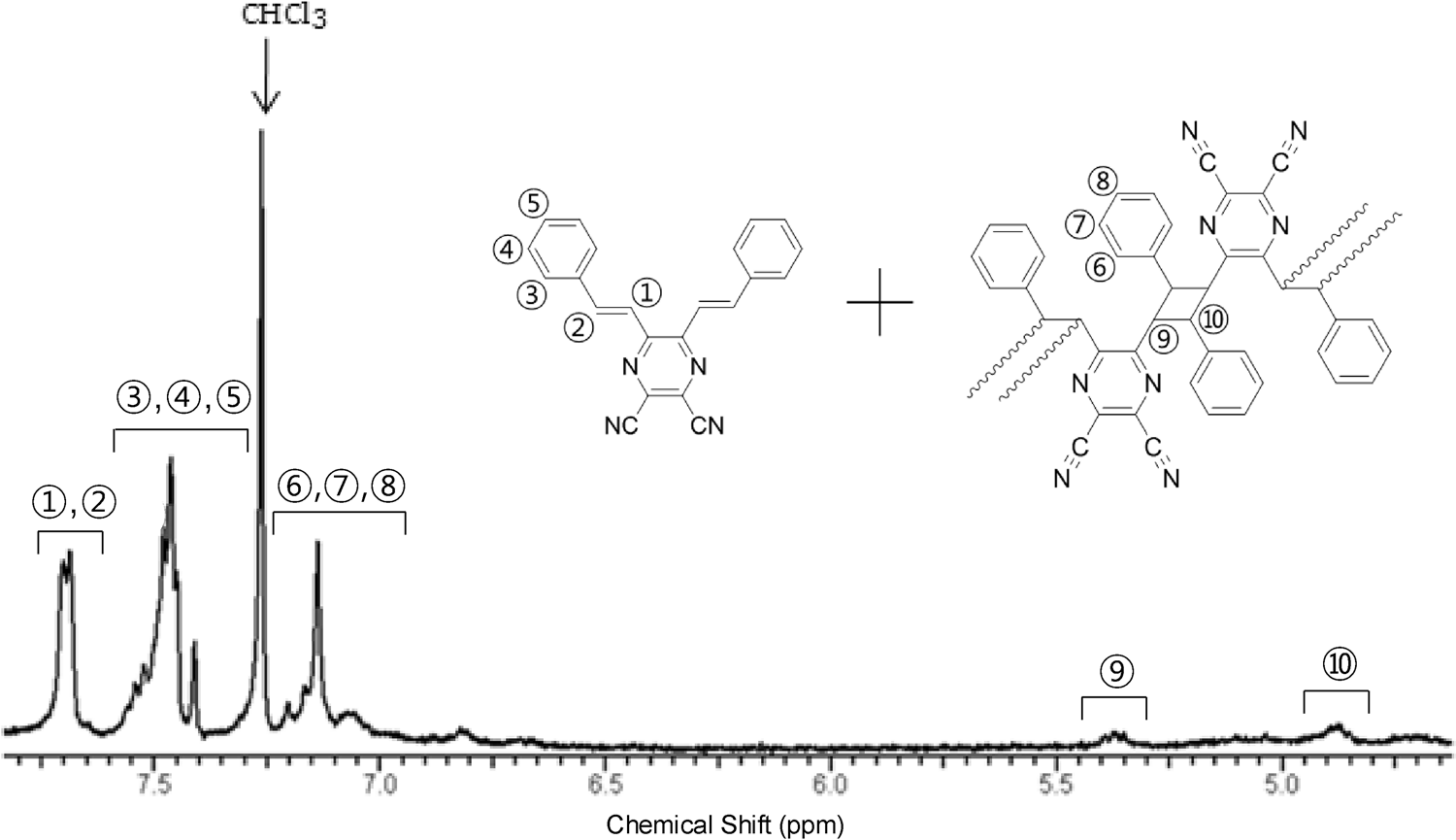
^1^H-NMR spectrum of RO-form in CDCl_3_.

On the other hand, the RO-form recovered from G-form by UV irradiation shows different ^1^H-NMR spectra; two new peaks (⑨ and ⑩) at ~ 5.37 and ~ 4.87 ppm, probably due to the presence of protons in cyclobutane, and other new peaks (⑥, ⑦, and ⑧) resulted from the phenyl rings attached to the cyclobutane (Fig. 6). This indicates that DSP underwent [2 + 2] photocycloaddition under UV irradiation, which produced oligomers as shown in the inset of Fig. 6. The ^1^H-NMR peak integration ratio of the original phenyl ring (③–⑤) to the phenyl ring attached to the cyclobutane (⑥–⑧) in the RO-form was 1.1:1.0, while the theoretical integration ratio of the ideal oligomer assumed to be fully reacted should be almost 0:1. This suggests that large amount of monomers still remained even after the photocycloaddition reaction. From this integration ratio, the conversion ratio from the monomer to the oligomer was determined to be ~ 47%. This means that both the oligomer and monomer of DSP coexist in the RO-form. The XRD result showed that the RO-form obtained from UV irradiation of G-form has same diffraction peaks as O-form. It implies that the crystal structure of RO-form is same as O-form. The photocycloaddition under UV irradiation allows the movement of the lattice (lattice slipping)^23^, which helps the crystal structure change from G- to O-form. Furthermore, the G-form stemmed from the grinding of RO-form shows only ^1^H-NMR peaks originated from the monomer but not oligomer (Fig. S2). The formation of the cyclobutane and conversion to the monomer of DSP can also be verified through FT-IR spectra. Figure S3 shows the chemical structure of RO- and G-forms. New absorption peaks appeared at 2850 and 2920 cm^−1^ in RO-form due to the aliphatic C–H stretching of the newly formed cyclobutane ring but disappeared after the re-grinding. This result confirms that the repeatable processes, the formation of cycloadduct in RO-form caused by UV irradiation and decomposition of cycloadduct into its monomer by the mechanical stress, took place during the G → RO → G transition process.

**Figure 7:**
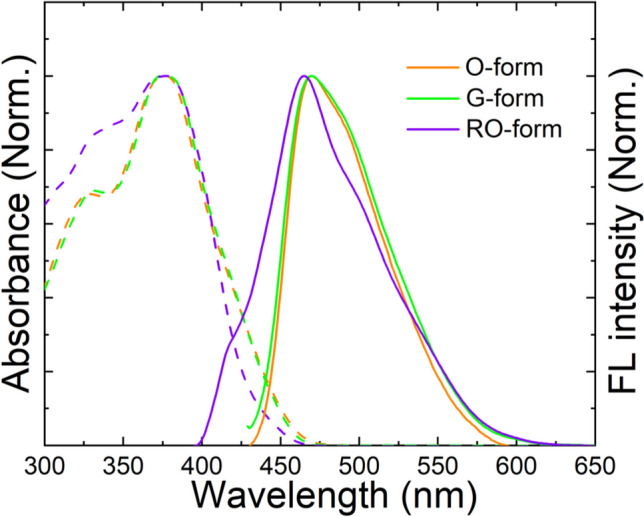
UV–visible absorption and FL emission spectra of O-, G- and RO-forms in solution (1.0 × 10^‒6^ M in CHCl_3_, excited at 365 nm).

A highly dilute solution can be considered as an ideal solution excluding intermolecular interactions; hence, it is possible to measure the intrinsic optical properties of molecules in the isolated state. Figure 7 shows the UV–visible absorption and FL emission spectra of three different crystals in a dilute solution (1.0 × 10^−6^ mol/L in CHCl_3_). As observed through the ^1^H- NMR results, the O- and G-forms showed almost the same absorption (maximum peak, λ_abs, max_ = 377 nm; shoulder peak_,_ λ_abs, shoulder_ = 327 nm) and FL emission (λ_abs, max_ = 470 nm) spectrum. However, the RO-form shows similar but slightly different UV–visible absorption and FL emission spectrum. The shoulder absorption around 330 nm increased, the FL maximum peak slightly shifted to a shorter wavelength, and a new small shoulder FL emission peak appeared at 420 nm. This is probably because the oligomer has a shorter conjugation length owing to the cyclobutane moiety compared to the monomer. This reconfirms the idea that the G-form underwent [2 + 2] photocycloaddition under UV irradiation, producing the RO-form as a mixture of oligomer and monomer.

**Figure 8:**
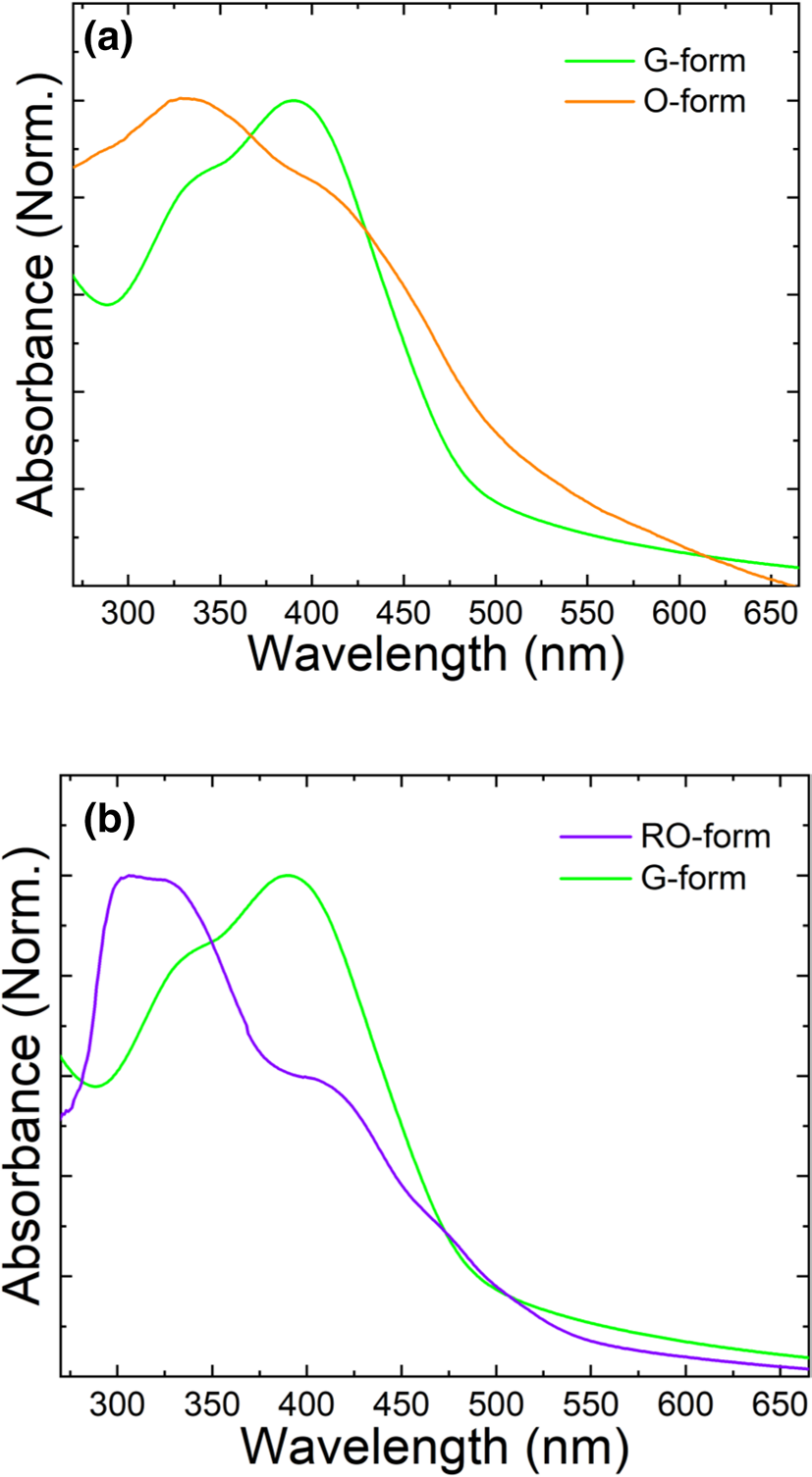
UV–visible absorption spectra of DSP crystals in the interconversion between (**a**) G- and O-forms and (**b**) RO- and G-forms.

Figure 8a shows the UV–visible absorption spectra of DSP crystals in the interconversion between the O- and G-forms. Unlike the peaks observed in the solution, quite different absorption peaks were observed. The G-form showed two absorption peaks at 330 nm and 390 nm. The absorption peak at the shorter wavelength came from the monomer without π-π intermolecular interaction as shown in Fig. 7 and the absorption peak at the longer wavelength came from molecular stacking structure in aggregate. The O-form also showed the absorption peaks at the shorter (330 nm) and the longer (405 nm and 460 nm) wavelength. The further bathochromic shift of absorption peak at the longer wavelength clearly proves that O-form have denser molecular stacking structure than G-form. These results should come from the difference molecular stacking structure of each crystal and supports well the idea that change of molecular stacking structure by external mechanical stimuli causes the difference in the optical properties, even though crystals have same chemical composition. It can be also clarified by the DSC results (Fig. S4). The G-form showed a very small but clear endothermic peak at 178.6 °C, due to a crystal-to-crystal transition of lattice contraction in addition to a sharp endothermic peak at 253.3 °C due to melting, while the O-form showed only a sharp endothermic peak at 258.2 °C^24^. As shown in Fig. 8b, the RO-form obtained by the UV irradiation of G-form showed a UV–visible absorption spectrum similar to the O-form. The absorption peak at 306 nm resulted from oligomers in RO-form, which have a shorter conjugation length due to a cyclobutane ring structure. Consequently, the G-to-RO phase transition by UV irradiation results from a change of molecular stacking structure due to lattice movement (lattice slipping) allowed by photocycloaddition in solid-state.

**Figure 9:**
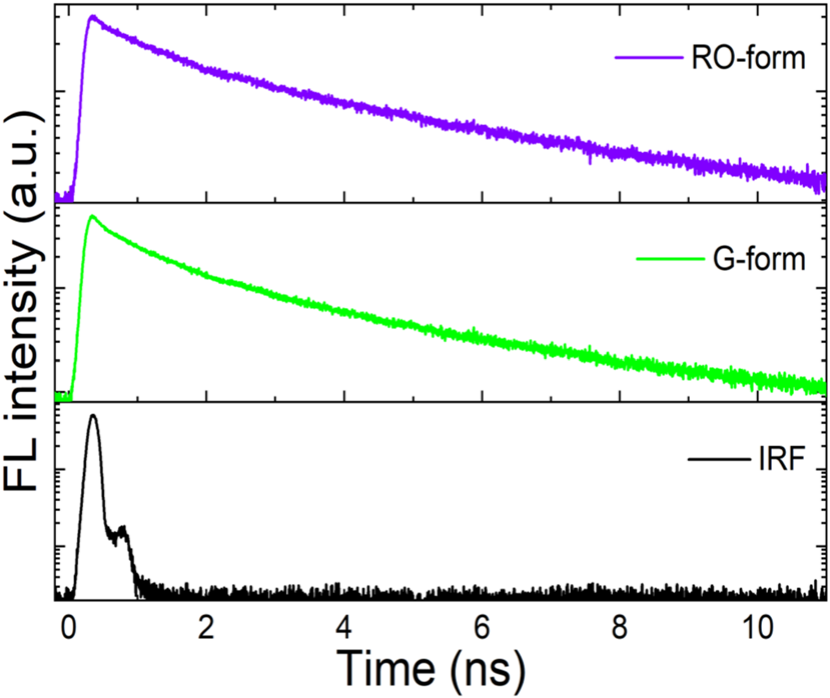
FL emission decay profiles of DSP crystals in G- and RO-forms.

Figure 9 shows the FL dynamics of DSP crystals in the G- and RO-forms. The FL emission decay was monitored at around the λ_FL, max_ of each crystal form. The FL emission decayed more slowly in the RO-form than in the G-form. The average exciton lifetime of the RO-form was 3.30 ns, 1.5 times longer than that (2.17 ns) of the G-form (Table S1). In addition, the fraction of long-lived species is also higher in the RO-form than that of the G-form. The longer FL lifetime of the RO-form can be attributed to the formation of excimer species in the excited state. The excimer energy state is readily induced as the degree of intermolecular stacking increases. On the other hand, a shorter FL lifetime of the G-form indicates the monomer emission with a lower degree of intermolecular stacking. This result well supports the hypothesis that RO-form have denser molecular stacking structure than G-form due to enhanced intermolecular π–π interaction.

As mentioned previously, a significant FL emission change of DSP crystal from orange to green was observed by mechanical grinding, resulting from crystal-to-crystal phase transition with different molecular stacking structure. This is a usual case in PCFL, and similar phenomena have been widely reported by other research groups^7–22^. However, in most cases, the original FL was restored by thermal annealing or recrystallization. As far as we know, no one has ever reported the restoration of original FL by UV irradiation so far. Thus, we have investigated the oligomer formation mechanism by UV irradiation in more detail to clarify the unusual restoration of PCFL. According to previous reports, the distance between the neighboring vinylene moieties in the G-form in X-ray analysis was 5.7 Å^23^. This does not satisfy the Schmidt rules that the distance between the functional groups should be < 4.2 Å for the photocycloaddition reaction^25–27^. However, the ^1^H-NMR spectrum clearly showed that DSP underwent [2 + 2] photocycloaddition under UV irradiation, producing oligomers in the RO-form. As proven by the Kaupp group, therefore, the present G-to-RO interconversion should be an exceptional case where the photocycloaddition occurs through reorganization during the UV irradiation despite a much longer distance relative to the effective distance^28–30^.

## Conclusion

In this study, a unique PCFL phenomenon has been investigated using a distyrylpyrazine derivative, DSP crystal. Two different crystal forms, G-form and O-form, with green and orange emission, respectively, were prepared separately by the recrystallization of DSP from different solvents. The O-form was readily transformed to the G-form by manual grinding. Subsequently, when exposed to UV light, the G-form was converted to the orange FL emission RO-form, whose FL emission is slightly different from the O-form. When the RO-form was ground again, the G-form was regenerated. This PCFL behavior between the G- and RO-forms was repeated over several ten times. The O (or RO)-to-G transformation by grinding can be ascribed to the change of degree of molecular stacking from dense molecular stacking structure to relatively loose molecular stacking structure, whereas the FL emission restoration (G-to-RO) through UV irradiation can be ascribed to a return to dense molecular stacking structure again due to lattice movement (lattice slipping) allowed by photocycloaddition in solid-state. The restoration of orange color FL emission by UV irradiation is an entirely new method, whereas the original FL emissions are commonly restored exclusively by heating or recrystallization. This will be a convenient and universal method for achieving PCFL. Our results will also be helpful in developing novel metachromatic fluorophores for further advanced applications.

### Materials

The 2,3-diisocyano-5,6-distyrylpyrazine (DSP) used in this study was synthesized following a literature method^23^. Solvents and reagents were purchased from Sigma-Aldrich.

### Characterization

^1^H-NMR spectra were obtained using CDCl_3_ as the solvent at 25 °C (AVANCE digital 400 NMR (Bruker)). Chemical shifts were referred to tetramethylsilane (TMS) at 0 ppm. FT-IR spectra were recorded by a JASCO FT/IR-4100 spectrometer equipped with a JASCO ATR (attenuated total reflectance) model PR0450-S. Differential scanning calorimetry (DSC, TA Instruments Q2000) was performed under pure nitrogen gas at heating rate of 10 °C min^−1^. UV-absorption spectroscopy was performed using a JASCO V-650 spectrophotometer. FL emission spectra were recorded using a JASCO FP-6500 spectrofluorometer at excitation wavelengths of 375 nm (solutions), 340 nm (G-form), and 390 nm (O-form, RO-form). The solid state FL quantum yields (FLQYs) were obtained relative to 9,10-diphenylanthracence in poly(methyl methacrylate) (PMMA) matrix (*F*_re_ = 0.83, 10^−3^ M). Fluorescence images of DSP crystals under UV irradiation at an excitation wavelength of 365 nm were recorded using a digital camera (Cannon PowerShot A2000 IS). The FL microscope images were recorded using a Nikon Eclipse E600 fluorescence optical microscope equipped with a Nikon DS-Fi1 digital camera and a super-high-pressure 100 W Hg lamp (OSRAM, HBO103W/2). The X-ray diffraction (XRD) experiments were performed using a Rigaku D/max-2500 diffractometer with Cu–Kα radiation (λ = 1.54 Å) at 40 kV and 100 mA. The FL emission decay of DSP crystals were investigated using a time-correlated single-photon counting (TCSPC) system. The second harmonic (SHG = 420 nm) of a tunable Ti:sapphire laser (Mira 900, Coherent) with ~ 150 fs pulse width and 76 MHz repetition rate was used as the excitation source. The FL emission was spectrally resolved using some collection optics and a monochromator (Acton, SP-2150i). A TCSPC module (PicoQuant, PicoHarp 300) with a MCP-PMT (Hamamatsu, R3809U-59) was used for ultrafast detection. The total instrument response function (IRF) for the FL decay was less than ~ 140 ps and the temporal time resolution was less than 10 ps. Deconvolution of the actual fluorescence decay and IRF was performed by using fitting software (FlouFit, PicoQuant) to deduce the time constant associated with each exponential decay.

## Supplementary Information

Supplementary Information.
