# Supplementary material for: Unusual piezochromic fluorescence of a distyrylpyrazine derivative crystals: phase transition through [2 + 2] photocycloaddition under UV irradiation
Source: Sci Rep. 2021 Feb 2;11:2762. doi: 10.1038/s41598-021-81562-4 (PMC7854636; doi:10.1038/s41598-021-81562-4)
Supplement: Supplementary file 1 — Supplementary Information. [file 41598_2021_81562_MOESM1_ESM.pdf]

Supporting Information

Unusual piezochromic fluorescence of a distyrylpyrazine derivative crystals: phase transition through [2+2] photocycloaddition under UV irradiation

Young-Jae Jin[a], Hyosang Park[a], Byung-Chun Moon[a], Jae Hong Kim[b,*], Wang-Eun Lee[c], Chang-Lyoul Lee[d,*], Giseop Kwak[a,*]

[a]Department of Polymer Science and Engineering, School of Applied Chemical Engineering, Kyungpook National University, 1370 Sankyuk-dong, Buk-ku, Daegu 702-701, Korea

[b] School of Chemical Engineering and Technology, Yeungnam University, 214-1, Dae-dong, Gyeongsan, Gyeongbuk 712-749, Korea

[c]Reliability Assessment Center for Chemical Materials, Korea Research Institute of Chemical Technology (KRICT), 141 Gajeong-ro, Yuseong-gu, Daejeon 305-600, Korea

[d]Advanced Photonics Research Institute (APRI), Gwangju Institute of Science and Technology (GIST), 1 Oryong-dong, Buk-gu, Gwangju, Korea

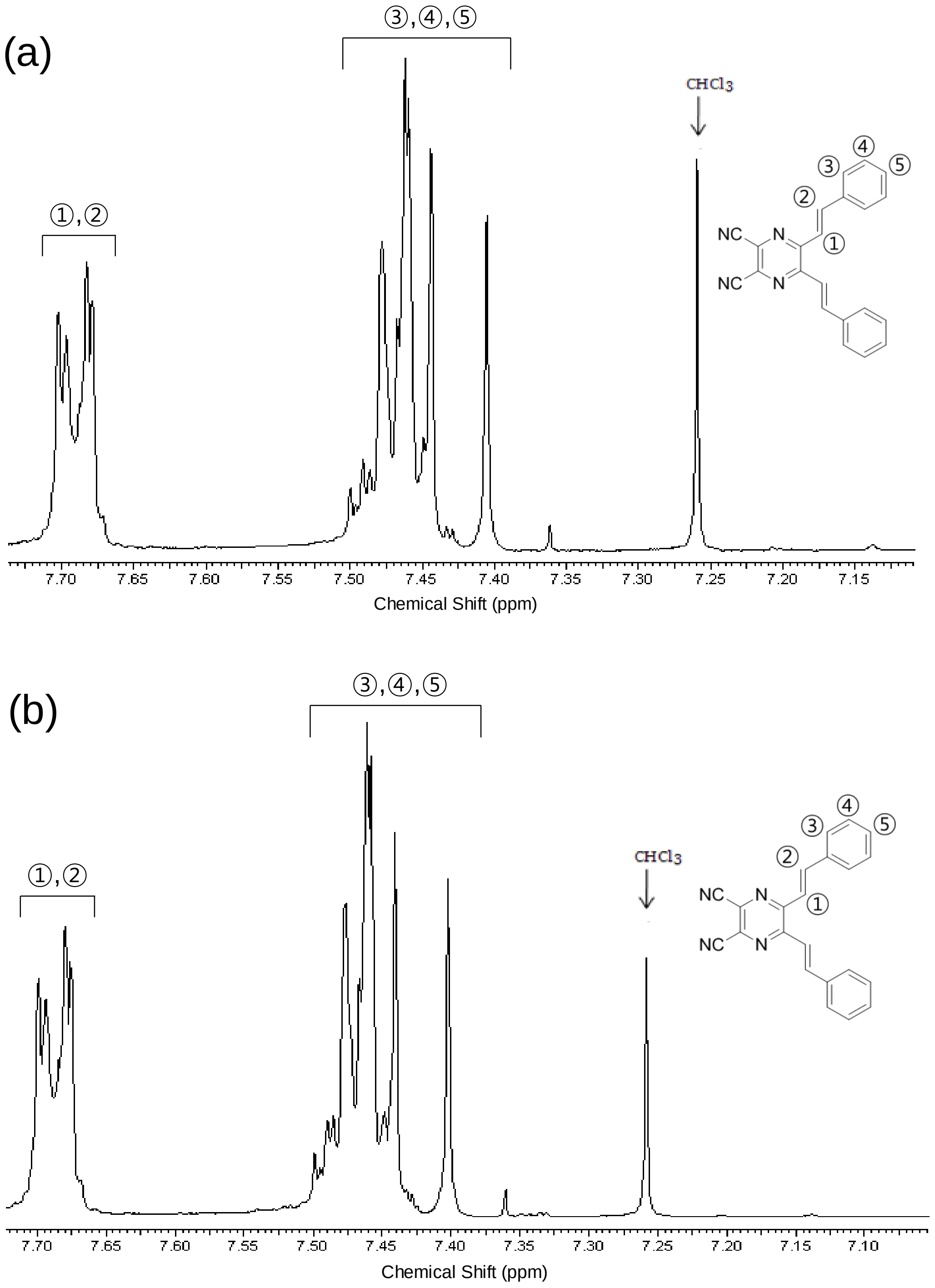

Figure S1. ^{1}H-NMR spectra of **(a)** O- and **(b)** G-forms in $CDCl_3$.

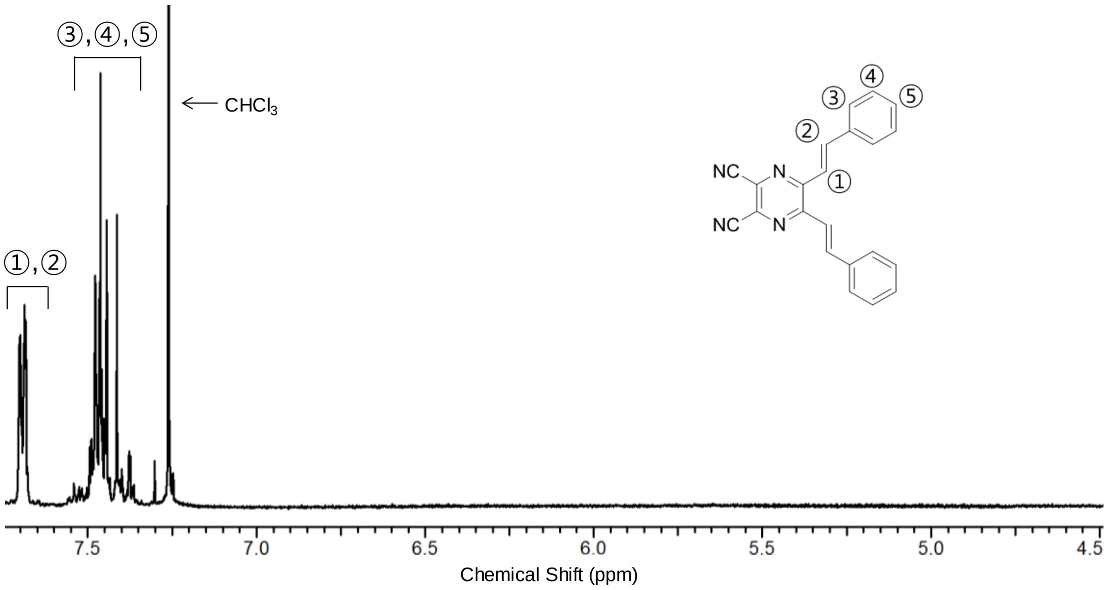

Figure S2. ^{1}H-NMR spectra of G-form recovered from the RO-form in $CDCl_3$.

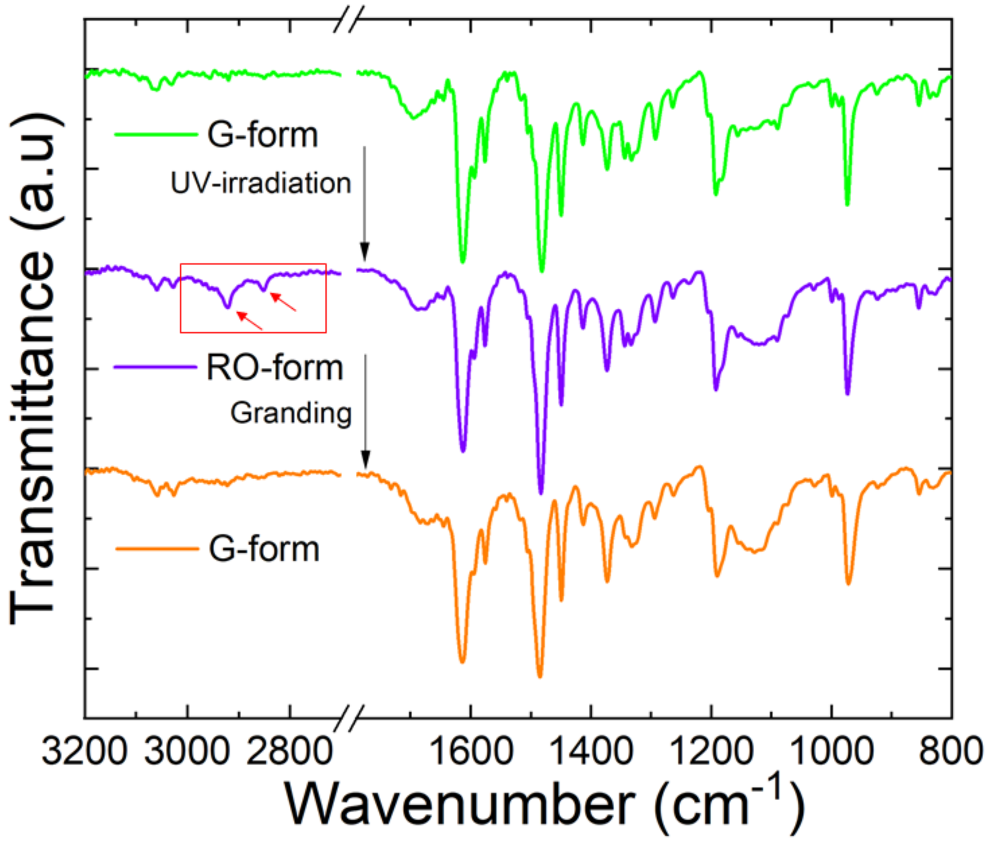

Figure S3. FT-IR spectra of DSP crystals during the G→RO→O form change.

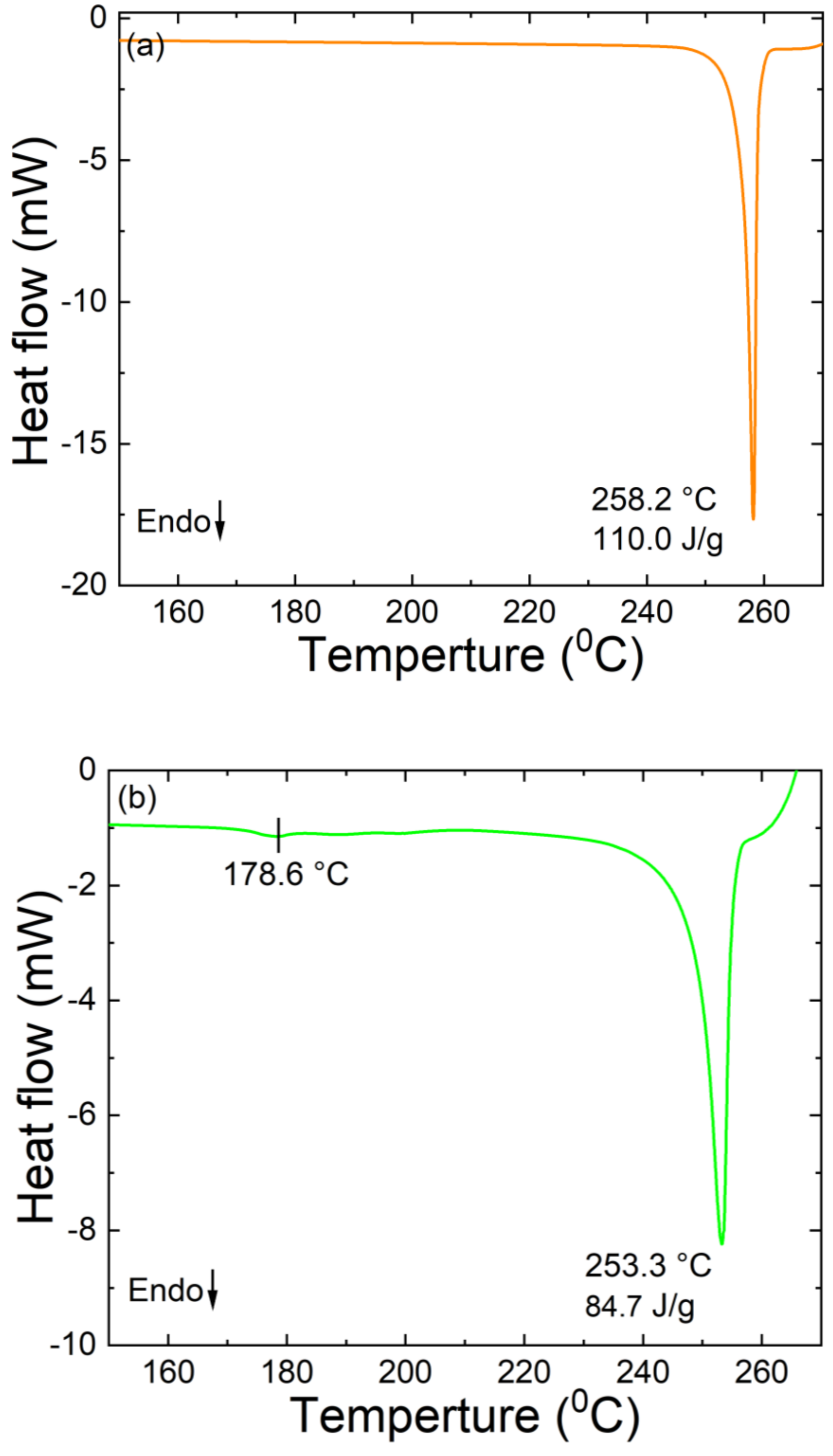

Figure S4. DSC thermograms of **(a)** O- and **(b)** G-forms during heating trace (heating rate = 10 ℃ min^{-1}).

Table S1. FL lifetimes of DSP crystals in G- and RO-forms

Crystal form	Monitoring wavelength (nm)	τ_1 (ns)	f_1	τ_2 (ns)	f_2	τ_{ave} (ns)[a]	χ^2[b]
RO-form	565	3.60	0.90	0.58	0.1	3.30	1.152
G-form	515	2.68	0.76	0.57	0.24	2.17	1.195

The FL decay curves were fitted a bi-exponential function to calculate the lifetime of DSP crystals. [a]The intensity-weighted average exciton lifetime (τ_{ave}) was $f_1\tau_1 + f_2\tau_2$, where f_1 and f_2 are fractional intensities and τ_1 and τ_2 are the lifetimes (ns). [b]χ^2 is the reduced chi-square.
